# The Role of Traditional Chinese Medicine Nursing for Stroke: An Umbrella Review

**DOI:** 10.1155/2021/9918687

**Published:** 2021-06-30

**Authors:** Caixia Hu, Xiaohui Qin, Richun Ye, Minqing Jiang, Yuhua Lu, Changting Lin

**Affiliations:** Department of Neurology, The Second Affiliated Hospital of Guangzhou University of Chinese Medicine (Guangdong Provincial Hospital of Chinese Medicine), Guangzhou, China

## Abstract

**Background:**

An increasing number of systematic reviews/meta-analyses (SRs/MAs) of clinical trials have begun to investigate the effects of traditional Chinese medicine (TCM) nursing in patients with stroke. To systematically appraise and synthesize these results, we conducted an overview of SRs/MAs.

**Methods:**

Eight databases from their inception to April 2020 were searched to include all SRs/MAs on TCM nursing for stroke. Methodological quality assessment was performed using Assessing the Methodological Quality of Systematic Reviews 2 (AMSTAR-2) and evidence quality assessment was performed using the Grading of Recommendations, Assessment, Development, and Evaluation (GRADE).

**Results:**

Eleven SRs/MAs regarding TCM nursing for stroke were included. The assessments with AMSTAR-2 indicated that the methodological quality of all included SRs/MAs was critically low. According to the evaluation results of GRADE, 10 (40%) outcomes were rated as critically low-quality evidence, 7 (28%) low-quality evidence, and 8 (32%) moderate-quality evidence. Descriptive analysis results showed that TCM nursing was effective for stroke.

**Conclusions:**

All included SRs/MAs suggested positive findings of TCM nursing for stroke, but the credibility of the results is limited. Studies with methodologically rigorous and adequately powered are still needed in this field.

## 1. Introduction

Stroke is the second leading cause of death and third leading source of disability worldwide [1]. It has been reported that the prevalent cases of stroke patients exceeded 79 million in 2016 [2]. According to the type of pathological process, stroke can be divided into hemorrhagic stroke and ischemic stroke types. The incidence of hemorrhagic stroke in China has decreased by 1.7% in the past 21 years, but the incidence of ischemic stroke has increased by 8.7% annually [3]. Among stroke survivors, a large number of physical, psychological, and social functions caused by stroke are often left behind, which has a great impact on the patient's daily life ability, social function, and quality of life, and brings great economic pressure to the family and society [4]. Therefore, the care of stroke patients has always been an area of focus for researchers. In addition to routine care, there are multifold health needs and concerns among patients with stroke. Many people are interested in complementary and alternative medicine.

Traditional Chinese medicine (TCM) nursing was established under the guidance of the basic theory of TCM [5]. According to a survey, 42% of Americans had experienced complementary and alternative medical treatments [6], to which traditional Chinese medicine (TCM) nursing techniques contribute a great proportion [7]. Based on preliminary literature search results, several systematic reviews (SRs)/meta-analyses (MAs) on TCM nursing for stroke have been published. These articles are considered evidence that appropriate knowledge can be provided for clinical decision-making. However, only high-quality SRs/MAs are reliable, while low‐quality articles increase the risk of misleading decisions, so the importance of strictly implementing and reporting SR/MAs cannot be overemphasized [8]. The aim of this overview was to evaluate the methodological quality and evidence quality of SRs/MAs in TCM nursing for stroke. This overview also intended to identify the deficiencies and provide recommendations for achieving high‐quality SRs/MAs.

## 2. Materials and Methods

The methodology of this overview was followed the guidelines stated in the Cochrane handbook and the method of high-quality overviews [9–11]. The protocol of this study was registered in PROSPERO (registration ID: CRD42021248912).

### 2.1. Inclusion and Exclusion Criteria

To be included, SRs/MAs had to meet the following criteria: (1) the definition of TCM nursing was guided by the Routine and Technical Operation Regulations of Nursing Care in TCM [5]. Eighteen non-invasive approaches were incorporated as TCM nursing techniques, namely, auricular plaster, moxibustion, acupoint massage, cupping, scrapping, TCM wet compress, TCM soaking, TCM medicated bath, TCM drug smearing, TCM dressing change, TCM acupoint application, TCM topical application, TCM ironing, TCM iontophoresis, TCM fumigation, TCM emotional nursing, TCM exercise nursing, and TCM dietary nursing [8]; (2) randomized controlled trial (RCT) on the effects of TCM nursing for stroke patients; (3) TCM nursing may have been implemented with routine care or alone; (4) subjects were diagnosed with WHO criteria using appropriate radiological methods. Exclusion criteria: duplicates, and reviews in which data cannot be extracted.

### 2.2. Search Strategy

Eight databases (PubMed, Embase, the Cochrane Library, Web of Science, China National Knowledge Infrastructure (CNKI), Wanfang Database, Chongqing VIP, and Chinese Biomedical Database) were searched from their inception to April 2020. Search keywords were TCM nursing, stroke, systematic review, and meta-analysis.  Table 1 shows the detailed search strategy for PubMed.

### 2.3. Eligibility Assessment and Data Extraction

After removing duplicates of the retrieved citations, two independent reviewers screened the titles and abstracts of potentially eligible articles to determine whether the articles met the eligibility criteria. After preliminary screening, full-text manuscripts of potential articles were retrieved and reviewed in detail according to eligibility criteria.

Data extractions from included reviews were extracted by two independent reviewers following the standardized data extraction forms. Study characteristics (e.g., first author, publication year, sample size, and stroke status), details of the intervention (including style of TCM nursing, duration, frequency, and time), methodological quality assessment tools, outcomes, and summery results were also extracted.

### 2.4. Methodological Quality Assessment

The methodology quality of the included SRs/MAs was independently evaluated by two reviews using the Assessing the Methodological Quality of Systematic Reviews 2 (AMSTAR-2) [12]. It has 16 items that require respondents to answer “yes,” “no,” or “partially yes.” Among these 16 items, seven are key items, and the remainder are non-key items. AMSTAR-2 categorizes the overall confidence on the results of the SRs/MAs into four levels: high, moderate, low, and critically low.

### 2.5. Evidence Quality Assessment

The evidence quality of the included SRs/MAs was independently evaluated by two reviews using the Grades of Recommendation, Assessment, Development, and Evaluation (GRADE) system [13]. Factors leading to the degradation of evidence include study limitations, inconsistent study results, inability to determine whether it is direct evidence, insufficient accuracy or wide confidence intervals, and publication bias.

## 3. Results

### 3.1. Search Outcome

**Figure 1:**
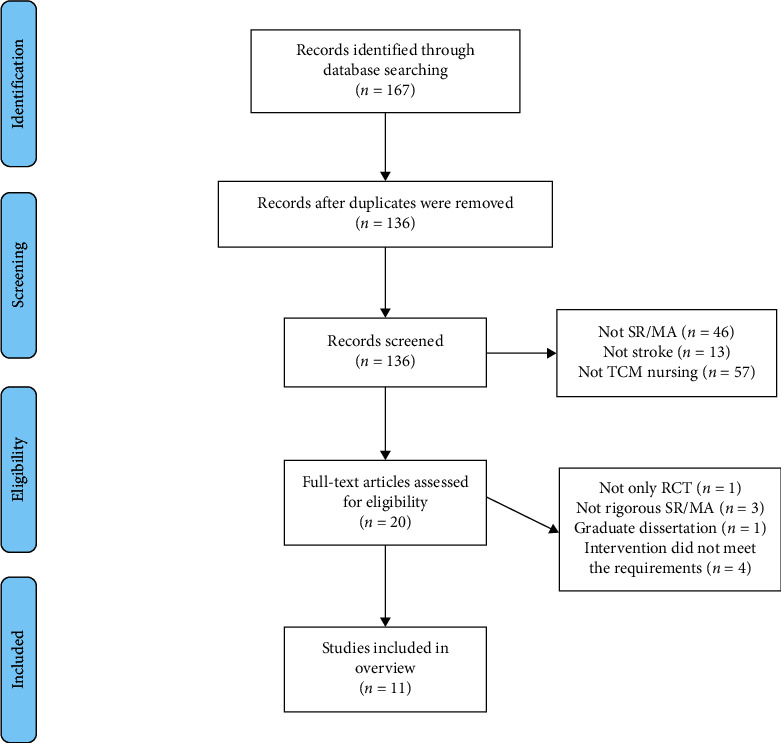
Literature selection procedure.

A total of 167 articles were identified and 31 duplicates were excluded. Subsequently, 116 records were excluded on the basis of their titles and abstracts. The remaining 20 articles were retrieved in full-text format for further assessment, and a further 9 articles were excluded. Finally, 11 [14–24] articles were reviewed and included in this overview. The flow chart describing the inclusion process and the reasons for exclusion is presented in Figure 1.

### 3.2. Study Characteristics

The descriptive characteristics of the included SRs/MAs are presented in Table 2. The SRs/MAs were published between 2015 and 2020 and included between 5 and 31 RCTs with sample sizes ranging from 458 to 2349 participants. All SRs/MAs were published by authors from China; one [14] was published in English, and ten [15–24] in Chinese. Patients were mainly those with stroke sequelae (e.g., poststroke limb dysfunction, poststroke dysphagia, poststroke depression, poststroke constipation, and post stroke shoulder-hand syndrome). The intervention measures were mostly TCM nursing (e.g., TCM exercise nursing, acupoint massage, TCM emotional nursing, auricular plaster, and TCM soaking) or TCM nursing plus routine nursing in the treatment group routine nursing in the control group. Ten SRs/MAs [14–23] used Cochrane risk of bias criteria to evaluate the quality of trails, and the remaining one [24] used Jadad scale. For original RCTs, especially those of better-quality studies, the key parameters of the different therapeutic paradigms used alone or in combination are listed in Table 3.

### 3.3. Methodological Evaluation

After evaluation of the methodological quality by the AMSTAR-2 tool, all included SRs/MAs were rated to be of critically low quality. The critical items that were most frequently lacking were item 2 (lack of protocol being registered before review commencement (*n* = 6, 100%)), item 4 (lack of search strategy for each database (*n* = 8, 72.7%)), item 7 (lack of list of excluded trails (*n* = 11, 100%)), and item 15 (lack of assessment of present and likely impact of publication bias (*n* = 2, 18.2%)). The breakdown of the AMSTAR-2 assessment is presented in Table 4.

### 3.4. Evidence Quality Evaluation

After evaluation of the evidence quality of the 25 outcome measures by the GRADE system, 8 (32%), 7 (28%), and 10 (40%) were rated to be of moderate, low, and critically low quality, respectively. The key factors affecting the evidence quality included the study limitations within the original RCTs, inconsistency, publication bias, and imprecision. Details of the GRADE evaluation are shown in Table 5.

### 3.5. Outcomes and Efficacy Evaluation

#### 3.5.1. Poststroke Limb Dysfunction

One review [14] analyzed the effectiveness of TCM exercise nursing for patients with poststroke limb dysfunction using subjective quantitative assessment scales, including FMA, BBS, ADL, SPPB, and NIHSS. According to the results, it was found that TCM exercise nursing combined with rehabilitation nursing was more effective than rehabilitation nursing alone.

#### 3.5.2. Poststroke Dysphagia

Two reviews [15, 19] analyzed the effectiveness of acupoint massage combined with rehabilitation nursing for patients with poststroke dysphagia using effective rate, WST score, and incidence of aspiration pneumonia. Of these outcomes, both the effective rate and WST score gave positive results, but no significant difference was found in terms of incidence of aspiration pneumonia.

#### 3.5.3. Poststroke Depression

Two reviews [16, 23] analyzed the effectiveness of TCM emotional nursing for patients with poststroke depression using HAMD score, and both of these two SRs/MAs concluded positive findings.

#### 3.5.4. Poststroke Constipation

Two reviews [17, 20] analyzed the effectiveness of TCM nursing for patients with poststroke constipation using effective rate, cure rate, and incidence of constipation. Both of these outcomes gave positive results.

#### 3.5.5. Poststroke Shoulder-Hand Syndrome

One review [18] analyzed the effectiveness of TCM soaking for patients with poststroke shoulder-hand syndrome using effective rate, FMA score, and BI score. According to the results, it was found that TCM soaking combined with rehabilitation nursing was more effective than rehabilitation nursing alone.

#### 3.5.6. Stroke Sequelae

Three reviews [21, 22, 24] analyzed the effectiveness of TCM nursing for patients with poststroke constipation using effective rate, FMA score, HAMD score, and ADL score. Both of these outcomes gave positive results.

## 4. Discussion

### 4.1. Summary of Main Findings

Eleven SRs/MAs on TCM nursing for stroke were included, published from 2015 to 2020. All studies were published within the past five years, meaning that TCM nursing has become a new research concern. This overview included 25 outcome measures, almost all of which reached positive conclusions. Although most of the included SRs/MAs showed that TCM nursing was beneficial for stroke, the authors did not want to draw firm conclusions because of the small size or the low methodological quality of the included trails. In addition, based on the evaluation results of AMSTAR-2 and GRADE, the methodological quality and the evidence quality of the included SRs/MAs are not satisfactory, indicating that the results of SRs/MAs may differ significantly from the real situation. Therefore, high-quality and large simple size studies are still needed to determine precisely the efficacy and safety of these stroke interventions.

### 4.2. Summary of Quality

The results of AMSTAR-2 evaluation show that each SRs/MAs has different deficiencies in terms of design, registration, data extraction, and analysis. All SRs/MAs did not include a preliminary design protocol, which may result in greater adjustments to the research process than expected, increase the risk of bias, and affect the rigor of SR/MA. Only two SRs/MAs provided specific search strategy, which may result in publication bias and affect the conclusion's reliability. All SRs/MAs did not provide a complete list of potential studies, which may affect the reliability of the results. The list of excluded articles provided could more strongly prove the rigor of the literature screening process. Only four SRs/MAs reported funding resources and stated the conflicts of interests, which may increase study reporting bias. Two SRs/MAs did not take published bias into account when the authors explain or discuss the results of the study and explain their possible sources, which may affect the authenticity of the final results. The above defects are the main reason for the very low quality of the included SR/MA methodology.

The results of GRADE evaluation indicate that the current quality of evidence is unsatisfactory. The key factors affecting the evidence quality included limitations, inconsistency, imprecision, and the possibility of publication bias. These evidence quality defects suggest that the conclusions of the SRs/MAs may be different from the true results and therefore do not provide a scientific basis for clinicians. The limitations of the original RCTs are the main factor in the decrease of the quality of all evidence. Of these original RCTs, most of them only mentioned randomization but did not describe the randomization method; most did not conceal the allocation; only a few mentioned blinding, and most of these used only single blinding. These common deficiencies limited the quality of the original RCTs and the evidence quality of the SRs/MAs.

### 4.3. Current Research Status and Implications for Future Research

TCM has thousands of years' history in China. Nursing science characterized by TCM, named TCM nursing, is a significant component of complementary and alternative medicine. Different from ordinary physiotherapy, TCM nursing shares the same theory and philosophy as TCM, and the advantages of TCM nursing include health education based on theories and techniques of TCM [5]. In China, the doctor of TCM mainly focuses on diagnosis and medication for disease and the application of invasive treatments such as acupuncture, while the nurse of TCM focuses mainly on health education and application of techniques of TCM [42]. In all TCM hospitals and wards of TCM in general hospitals, there are professional nurses engaging in TCM; TCM nursing plays a significant role in the Chinese healthcare system [43]. Higher education in TCM nursing starts relatively late in China, with undergraduate education being only offered in three TCM universities in 1999 and postgraduate education in TCM nursing being only offered in four universities in 2006, resulting in a relative lack of TCM nursing professionals [44]. Up to now, TCM nursing does not have a mature and complete theoretical system and practice standards [43]. Hence, when making clinical decisions, nurses often use other sources of evidence, such as their own experiences [45]. This dilemma is currently faced by TCM nursing techniques.

Evidence is the top priority for a smart medical decision. The concept of evidence-based medicine changes nurses' practice from experience-based to evidence-based, guiding nurses to retrieve and make use of the best evidence available. This concept would be beneficial in the literature review of TCM nursing research, health education, and standardization of TCM nursing techniques, and may solve the dilemma faced by TCM nursing to a certain extent [43]. As sources of the highest level of evidence for evidence-based medicine, SRs/MAs have been widely used in recent years [11]. According to the evaluation of this overview, common deficiencies are identified, and recommendations for achieving high‐quality SRs/MAs and RCTs are still need. For SRs/MAs, advance protocol registration, comprehensive literature search, list of excluded literatures and description of the reasons, reasonable explanation of publication bias, and conflict of interest statement should be focused on to improve the review quality. For RCTs, well-designed and implemented RCTs are considered the gold standards for evaluating interventions to minimize or avoid bias [11]. Therefore, high-quality RCTs with large sample sizes should give more attention to randomization, blinding, and allocation concealment, which could result in more reliable evidence.

### 4.4. Limitations

This study has certain limitations to be acknowledged. First, the stroke status and time post onset of the enrolled patients were not completely consistent, which may exaggerate or decrease the efficacy of TCM nursing and affect the robustness of the study conclusion. The prognosis of patients with different stroke status and time after onset is significantly different. Second, the protocols of TCM nursing are also diverse, including differences in TCM nursing techniques, stimulation methods, treatment duration, and number of treatments, which may result in a source of heterogeneity for the included reviews. Third, measurement scales used in the enrolled RCTs included SPBB, BBS, SAS, SDS, WST, FMA, etc., all of which were subjective clinical assessment tools based on the observation of the assessors. In addition, the most commonly used measure to assess the efficacy of TCM nursing for stroke was effective rate; however, it was not internationally recognized. Thus, the use of these indicators in the assessment of treatment effects may be inaccurate. Fourth, although objective criteria are established based on the evaluation of relevant literature, there may still be some subjectivity in the evaluation process. Finally, based on the current results, this study cannot draw firm conclusions for the use of TCM nursing for stroke. Further rigorous, comprehensive SRs/MAs and RCTs that adhere to the guidelines are required to provide robust evidence for definitive conclusions.

## 5. Conclusion

All included SRs/MAs suggested positive findings of TCM nursing for stroke, but the credibility of the results is limited owing to the summary evidence of this overview. The methodological quality and evidence quality of the included studies were generally low. The practice of using the widely accepted tools like AMSTAR-2 and GRADE for designing, reporting, and assessing of SRs/MAs needs to be encouraged and advocated, hereby providing more convincing evidence based on their findings.

## Figures and Tables

**Table 1:** Search strategy for the PubMed database.

Query	Search term
#1	Cerebrovascular disorders [mesh] OR stroke [mesh] OR brain infarction [mesh] OR cerebral hemorrhage [mesh]
#2	Cerebrovascular disorder*∗*[title/abstract] OR stroke*∗*[title/abstract] OR brain infarction*∗*[title/abstract] OR cerebral hemorrhage*∗*[title/abstract] OR intracranial vascular disease*∗*[title/abstract] OR cerebrovascular disease*∗*[title/abstract] OR brain vascular disorder*∗*[title/abstract] OR cerebrovascular occlusion*∗*[title/abstract] OR cerebrovascular insufficiency*∗*[title/abstract] OR cerebrovascular accident*∗*[title/abstract] OR cerebrovascular apoplexy [title/abstract] OR brain vascular accident*∗*[title/abstract] OR apoplexy [title/abstract] OR anterior cerebral circulation Infarction[title/abstract] OR cerebrum hemorrhage*∗*[title/abstract] OR intracerebral hemorrhage*∗*[title/abstract] OR brain hemorrhage [title/abstract]
#3	#1 OR #2
#4	Traditional Chinese medicine [mesh]
#5	Traditional Chinese medicine[title/abstract] OR Chinese medicine [title/Abstract] OR Zhong Yi Xue [title/abstract] OR TCM [title/abstract] OR herbal drugs[title/abstract] OR auricular[title/abstract] OR moxibustion [title/Abstract] OR massage [title/abstract] OR tuina [title/abstract]
#6	#4 OR #5
#7	Nursing [mesh]
#8	Nursing [title/abstract] OR nurse*∗*[title/Abstract] OR nurse-led [title/Abstract] OR nursing care [title/Abstract] OR nursing intervention [title/abstract]
#9	#7 OR #8
#10	Meta-analysis as topic [mesh]
#11	Systematic review [title/abstract] OR meta-analysis [title/abstract] OR meta-analyses [title/abstract]
#12	#10 OR #11
#13	#3 AND #6 AND #9 AND #12

**Table 2:** Characteristics of the included reviews.

Authors, reference no., year	Country	Stroke status	Trails (simple size)	Treatment intervention	Control intervention	Quality assessment tool	Overall conclusion
Li et al. [14], 2017	China	Poststroke limb dysfunction	31 (2349)	TCM exercise nursing; TCM exercise nursing + rehabilitation nursing	Rehabilitation nursing	Cochrane criteria	Current evidence showed that TCM exercise nursing produced positive effects on limb motor function, balance function, activity of daily living ability and neurological impairment among stroke patients. More large-scale, high-quality, multiple center RCTs are required to further verify above conclusions in the future.

Wang et al. [15], 2020	China	Poststroke dysphagia	10 (930)	Acupoint massage + rehabilitation nursing	Rehabilitation nursing	Cochrane criteria	According to the current literatures, acupoint massage could improve swallowing function in patients with post‐stroke dysphagia.

Yue and Li [16], 2019	China	Poststroke depression	5 (458)	TCM emotional nursing	Psychological nursing	Cochrane criteria	Routine nursing combined with TCM emotional nursing for patients with poststroke depression can effectively improve depressive symptoms.

Li et al. [17], 2019	China	Poststroke constipation	10 (1083)	TCM nursing + routine nursing	Routine nursing	Cochrane criteria	The effect of TCM nursing for poststroke constipation is better than that of the control group. However, due to fewer studies included in the analysis and the overall quality being low, more large samples and high-quality clinical research are needed to further confirm the efficacy of TCM nursing on stroke patients with constipation.
Wu [18], 2018	China	Poststroke shoulder-hand syndrome	6 (508)	TCM soaking + rehabilitation nursing	Rehabilitation nursing	Cochrane criteria	Present evidences showed that alternating TCM soaking combined with rehabilitation nursing on shoulder hand syndrome after stroke can improve the effective rate of treatment, upper limb motor function, and daily living ability of patients. But it needs to be verified by more high-quality studies

Wu et al. [19], 2018	China	Poststroke dysphagia	14 (1182)	Acupoint massage + rehabilitation nursing	Rehabilitation nursing	Cochrane criteria	The current evidence indicates that acupoint massage combined with rehabilitation nursing is beneficial to poststroke dysphagia. But more high-quality studies are needed to verify the above conclusion.

Wang et al. [20], 2017	China	Poststroke constipation	7 (579)	Auricular plaster + routine nursing	Routine nursing	Cochrane criteria	Auricular plaster is more effective than routine nursing in the treatment of poststroke constipation, and auricular plaster may reduce the occurrence of side effects of Western medicine.

Yuan and Wang [21], 2016	China	Stroke sequelae	14 (1453)	TCM nursing + routine nursing	Routine nursing	Cochrane criteria	TCM nursing has the effects of improving the therapeutic effect, relieving pain, improving depressive symptoms, improving self-care ability, and restoring motor function in patients with stroke sequelae.

Zhang and Liu [22], 2016	China	Stroke sequelae	8 (898)	TCM nursing + routine nursing	Routine nursing	Cochrane criteria	TCM nursing has a good effect on the rehabilitation of stroke patients. More large-scale, high-quality, multiple center RCTs are required to further verify above conclusions in the future.

Liang et al. [23], 2015	China	Poststroke depression	7 (664)	TCM emotional nursing + routine nursing	Routine nursing	Cochrane criteria	TCM emotional nursing can improve the depressive symptoms of patients with poststroke depression.
Peng et al. [24], 2015	China	Stroke sequelae	8 (663)	TCM nursing	Routine nursing	Jadad	TCM nursing may be superior to routine nursing for stroke patients. More large-scale, high-quality, multiple center RCTs are required to further verify above conclusions in the future.

**Table 3:** Characteristics of the analyzed trials.

Authors, year, reference no.	Patients characteristic (intervention E/control C)	Stroke status	Time after onset	Intervention (intervention E/control C)	Treatment duration	Outcomes
Taylor-Piliae and Coull, 2012, [25]	E (*n* = 13) mean age (SD): 72.8 ± 10.1; C (*n* = 12) mean age (SD): 64.5 ± 10.9	Poststroke limb dysfunction	E: 58.3 m E: 53.9 m	E: Tai Chi; C: routine nursing + weekly phone calls	60-minute class, 3 times per week, for 12 weeks.	SPPB, 2-minute step test

Au-Yeung et al., 2009, [26]	E (*n* = 59) mean age (SD): 61.7 ± 10.5; C (*n* = 55) mean age (SD): 65.9 ± 10.7	Poststroke limb dysfunction	E: 54.1 ± 79.2 m, E: 64.2 ± 106.4 m	E: Tai Chi; C: general physical therapy	Each week, 1 hour of group practice was supplemented by 3 hours of self-practice, for 12 weeks.	TUGT

Wang et al., 2013, [27]	E (*n* = 34) mean age (SD): 62.6 ± 5.7; C (*n* = 34) mean age (SD): 63.3 ± 6.0	Poststroke limb dysfunction	E: 25.31 ± 21.40 m, C: 15.07 ± 8.51 m	E: Tai Chi + routine rehabilitation exercise; C: routine rehabilitation	20∼30-minute class, 2 times per day, 10 times per week, for 6 weeks.	FMA-L, BBS, MBI

Huang 2016, [28]	E (*n* = 8) mean age (SD): 65.00 ± 6.16; C (*n* = 8) mean age (SD): 63.63 ± 7.37	Poststroke limb dysfunction	E: 15.13 ± 7.30 m, C: 18.63 ± 31.47 m	E: Tai Chi; C: routine exercise at home + phone calls or home follow-up	60 min per day, 2 times per week, for 24 weeks.	BBS, TUGT

Wang, 2016, [29]	E (*n* = 25) mean age (SD): 60.48 ± 8.29; C (*n* = 25) mean age (SD): 60.92 ± 10.07	Poststroke limb dysfunction	E: 5.50 ± 2.09 m, C: 5.08 ± 1.56 m	E: Tai Chi; C: routine rehabilitation therapy	30 min per day, 5 times per week, for 8 weeks.	BBS, MBI, TUGT

Lv, 2012, [30]	E (*n* = 30) mean age (SD): 66.13 ± 5.81; C (*n* = 30) mean age (SD): 65.2 ± 5.17	Poststroke constipation	Unclear	E: auricular plaster + Routine nursing; C: routine nursing	4 min per acupoint, 4 times per day, for 2 weeks.	Effective rate, number of bowel sounds

Yan, 2012, [31]	E (*n* = 29) mean age (SD): 64.49 ± 8.56; C (*n* = 28) mean age (SD): 66.82 ± 9.90	Poststroke dysphagia	E: 36.03 ± 14.70 d, C: 35.21 ± 17.48 d	E: acupoint massage + routine rehabilitation; C: routine rehabilitation	20 min each, 2 times per day, for 4 weeks.	Effective rate, WST

Wei, 2016, [32]	E (*n* = 100) mean age (SD): 58.35 ± 9.52; C (*n* = 100) mean age (SD): 57.89 ± 9.91	Poststroke dysphagia	E: 25.21 ± 5.18 d, C: 24.98 ± 5.13 d	E: acupoint massage + routine rehabilitation; C: routine rehabilitation	15 minutes each, 3 times per day, for 4 weeks.	Effective rate, WST
Zhao, 2011, [33]	E (*n* = 30) mean age (SD): 64.93 ± 8.80; C (*n* = 30) mean age (SD): 65.03 ± 8.64	Poststroke dysphagia	E: 58.57 ± 15.08 d, C: 61.33 ± 13.61 d	E: acupoint massage + routine rehabilitation; C: routine rehabilitation	5 min per acupoint, 2 times per day, for 4 weeks.	Effective rate, WST

Yao et al., 2012, [34]	E (*n* = 15) mean age (SD): 67.77 ± 10.24; C (*n* = 15) mean age (SD): 63.33 ± 9.48	Poststroke dysphagia	E: 26.50 ± 25.07 d, C: 28.43 ± 26.63 d	E: acupoint massage + routine rehabilitation; C: routine rehabilitation	2 min per acupoint, 20–30 min each, 1 time per day, for 2 weeks.	MAS, SAS, SDS

Hu, 2013, [35]	E (*n* = 32) mean age(SD): 60.5 ± 9.8; C (*n* = 32) mean age (SD): 62.2 ± 8.9	Poststroke constipation	E: 134.00 ± 1.26 d, C: 129.00 ± 1.35 d	E: auricular plaster + routine nursing; C: routine nursing	1 min per acupoint, 20–30 min each, 3-4 times per day, for 4 weeks.	SAS, SDS

Yu et al., 2014, [36]	E (*n* = 35) mean age (SD): 41.3 ± 7.28; C (*n* = 33) mean age (SD): 43.3 ± 6.89	Poststroke constipation	Unclear	E: auricular plaster + routine nursing + health education; C: routine nursing + health education	3 min per acupoint, 3 times per day, for 15 days.	Effective rate

Ji et al., 2015, [37]	E (*n* = 40) mean age (SD): 61.3 ± 8.6; C (*n* = 40) mean age (SD): 59.6 ± 9.7	Poststroke constipation	Unclear	E: auricular plaster + routine nursing; C: routine nursing	3 min per acupoint, 3 times per day, for 2 weeks.	Effective rate

Cai and Zhang, 2015, [38]	E (*n* = 28) mean age(SD): 56 ± 20; C (*n* = 28) mean age(SD): 57 ± 20	Poststroke constipation	E: 78.6 ± 77.2 d, C: 75.6 ± 78.8 d	E: auricular plaster + routine nursing; C: routine nursing	2 min per acupoint, 3 times per day, for 4 weeks.	Effective rate

Guo et al., 2011, [39]	E (*n* = 60) mean age (SD): 63.1 ± 3.2; C (*n* = 60) mean age (SD): 61.1 ± 2.6	Poststroke shoulder-hand syndrome	E: 1.7 ± 0.2 m, C: 1.5 ± 0.2 m	E: TCM soaking + routine rehabilitation exercise; C: routine rehabilitation exercise	First soak in hot water for 15 min, then soak in cold water for 15 min. 2 times per day, for 3 weeks.	Effective rate, VAS

Zhang et al., 2014, [40]	E (*n* = 40) mean age (SD): 53.61 ± 8.71; C (*n* = 40) mean age (SD): 54.21 ± 7.23	Poststroke shoulder-hand syndrome	2∼5 m	E: TCM soaking + routine rehabilitation exercise; C: routine rehabilitation exercise	First soak in cold water for 10 min, then soak in hot water for 10 min, and finally cool the blister for 10 min. 1 time per day, for 4 weeks.	VAS, FMA

Qu, 2013, [41]	E (*n* = 50) mean age (SD): 59.4 ± 1.5; C (*n* = 50) mean age (SD): 58.3 ± 1.4	Poststroke depression	Unclear	E: TCM emotional nursing + routine nursing; C: routine nursing	For 4 weeks.	Effective rate

SPBB: short physical performance battery; TUGT: timed up and go test; BBS: Berg balance scale; MBI: modified Barthel index; SAS: self-rating anxiety scale; SDS: self-rating depression scale; WST: water swallowing test; MAS: motor assessment scale; VAS: visual analog scale; FMA: Fugl–Meyer scale.

**Table 4:** Result of the AMSTAR-2 assessments.

Authors, reference no., year	AMSTAR-2																Quality
	Q1	Q2	Q3	Q4	Q5	Q6	Q7	Q8	Q9	Q10	Q11	Q12	Q13	Q14	Q15	Q16	
Li et al. [14], 2017	Y	PY	Y	Y	Y	Y	N	Y	Y	N	Y	Y	Y	Y	Y	N	CL
Wang et al. [15], 2020	Y	PY	Y	PY	Y	Y	N	Y	Y	N	Y	Y	Y	Y	Y	N	CL
Yue and Li [16], 2019	Y	PY	Y	PY	Y	Y	N	Y	Y	N	Y	Y	Y	Y	N	N	CL
Li et al. [17], 2019	Y	PY	Y	PY	Y	Y	N	Y	Y	Y	Y	Y	Y	Y	Y	Y	CL
Wu et al. [18], 2018	Y	PY	Y	Y	Y	Y	N	Y	Y	N	Y	Y	Y	Y	Y	N	CL
Wu et al. [19], 2018	Y	PY	Y	Y	Y	Y	N	Y	Y	N	Y	Y	Y	Y	Y	N	CL
Wang et al. [20], 2017	Y	PY	Y	PY	Y	Y	N	Y	Y	Y	Y	Y	Y	Y	N	Y	CL
Yuan and Wang [21], 2016	Y	PY	Y	PY	Y	Y	N	Y	Y	Y	Y	Y	Y	Y	Y	Y	CL
Zhang and Liu [22], 2016	Y	PY	Y	PY	Y	Y	N	Y	Y	N	Y	Y	Y	Y	Y	N	CL
Liang et al. [23], 2015	Y	PY	Y	PY	Y	Y	N	Y	Y	N	Y	Y	Y	Y	Y	N	CL
Peng et al. [24], 2015	Y	PY	Y	PY	Y	Y	N	Y	Y	Y	Y	Y	Y	Y	Y	Y	CL

Y: yes; PY: partial yes; N: no; CL: critically low; L: low; H: high.

**Table 5:** Certainty of evidences of SRs/MAs included.

Authors, reference no., year	Outcomes	Limitations	Inconsistency	Indirectness	Imprecision	Publication bias	Relative effect (95% CI)	Quality
Li et al. [14], 2017	FMA score	−1	−1	0	0	−1	SMD 1.21 (0.66, 1.77)	CL
	BBS score	−1	−1	0	0	−1	SMD 2.07 (1.52, 2.62)	CL
	ADL score	−1	−1	0	0	0	MD 15.60 (7.57, 23.63)	CL
	SPPB score	−1	0	0	−1	−1	MD −0.46 (−1.28, 0.36)	CL
	NIHSS score	−1	0	0	−1	−1	MD −2.57 (−3.14, −2.00)	CL

Wang et al. [15], 2020	WST score	−1	0	0	0	0	SMD 1.58 (1.37, 1.78)	M
	Effective rate	−1	0	0	0	0	RR 1.36 (1.26, 1.47)	M
	Incidence of aspiration pneumonia	−1	0	0	−1	−1	RR 0.40 (0.16, 0.99)	CL

Yue and Li [16], 2019	HAMD score	−1	−1	0	0	0	SMD −1.02 (−1.50, −0.55)	L

Li et al. [17], 2019	Effective rate	−1	0	0	0	0	OR 3.87 (2.43, 6.17)	M
	Incidence of constipation	−1	0	0	0	0	OR 0.23 (0.13, 0.42)	M

Wu et al. [18], 2018	Effective rate	−1	0	0	0	0	OR 3.74 (2.18, 6.41)	M
	FMA score	−1	0	0	−1	−1	MD 12.14 (10.75, 13.52)	CL
	BI score	−1	−1	0	−1	−1	SMD 2.68 (1.94, 3.43)	CL

Wu et al. [19], 2018	Effective rate	−1	−1	0	0	0	RR 1.27 (1.16, 1.39)	L
	WST score	−1	−1	0	0	0	MD −0.72 (−0.94, −0.50)	L

Wang et al. [20], 2017	Effective rate	−1	0	0	0	0	RR 1.49 (1.33, 1.67)	M
	Cure rate	−1	0	0	0	0	RR 1.97 (1.44, 2.71)	M

Yuan and Wang [21], 2016	Effective rate	−1	0	0	0	0	OR 4.22 (2.93, 6.08)	M
	FMA score	−1	−1	0	0	0	WMD 11.56 (3.85, 19.26)	L
	HAMD score	−1	0	0	−1	−1	WMD −6.95 (−8.94, −4.96)	CL
	ADL score	−1	−1	0	0	0	WMD 12.54 (2.60, 22.48)	L

Zhang and Liu [22], 2016	Effective rate	−1	0	0	0	−1	OR 3.31 (2.20, 4.97)	L
Liang et al. [23], 2015	HAMD score	−1	−1	0	0	−1	SMD −1.03 (−1.42, −0.64)	CL
Peng et al. [24], 2015	Effective rate	−1	0	0	0	−1	OR 7.44 (4.40, 12.58)	L

RR: risk ratio; OR: odds ratio; SMD: standardized mean difference; WMD: weighted mean difference; MD: mean difference. VL: very low; L: low; M: moderate; H: high; BBS: Berg balance scale; SPBB: short physical performance battery; WST: Water swallowing test; FMA: Fugl–Meyer scale; ADL: activity of daily living; BI: Barthel index; MMSE: NIHSS: National Institute of Health Stroke Scale; HAMD: Hamilton depression scale.

## Data Availability

The datasets used in the present review are available from the corresponding author on reasonable request.

## Abbreviations

TCM: Traditional Chinese medicine
SR: Systematic review
MA: Meta-analysis
AMSTAR-2: Assessing the methodological quality of systematic reviews 2
GRADE: Grading of recommendations, assessment, development, and evaluation
RCTs: Randomized clinical trials
CNKI: China national knowledge infrastructure
BBS: Berg balance scale
SPBB: Short physical performance battery
WST: Water swallowing test
FMA: Fugl–Meyer scale
ADL: Activity of daily living
BI: Barthel index
NIHSS: National Institute of Health stroke scale
HAMD: Hamilton depression scale.

## References

[1] Feigin V. L., Norrving B., Mensah G. A. (2017). Global burden of stroke. *Circulation Research*.

[2] GBD 2016 Disease and Injury Incidence and Prevalence Collaborators (2017). Global, regional, and national incidence, prevalence, and years lived with disability for 328 diseases and injuries for 195 countries, 1990–2016: a systematic analysis for the Global Burden of Disease Study 2016. *Lancet*.

[3] Zhao D., Liu J., Wang W. (2008). Epidemiological transition of stroke in China. *Stroke*.

[4] Hochstenbach J., Donders R., Mulder T., Limbeek J. V., Schoonderwaldt H. (1996). Long-term outcome after stroke. *International Journal of Rehabilitation Research*.

[5] China Association of Chinese Medicine (2006). *Routine and Technical Operation Regulations of Nursing Care in TCM*.

[6] Bunk S. (2001). Mainstreaming CAM: the unconventional cancer therapy boom challenges researchers to improve studies. (News). *The Scientist*.

[7] Wang J. J., Wang L. J., Bian X. M. (2009). The advantages and prospect of Chinese medical nursing skills. *Journal of Traditional Chinese Medicine Management*.

[8] Yang M., Jiang L., Wang A., Xu G. (2017). Epidemiology characteristics, reporting characteristics, and methodological quality of systematic reviews and meta-analyses on traditional Chinese medicine nursing interventions published in Chinese journals. *International Journal of Nursing Practice*.

[9] Huang J., Qin X., Shen M., Huang Y. (2020). An overview of systematic reviews and meta-analyses on acupuncture for post-stroke aphasia. *European Journal of Integrative Medicine*.

[10] Huang J., Shen M., Qin X., Huang Y. (2020). Effectiveness of auricular acupuncture for insomnia: an overview of systematic reviews. *Evidence-Based Complementary and Alternative Medicine*.

[11] Huang J., Shen M., Qin X., Guo W., Li H. (2020). Acupuncture for the treatment of tension-type headache: an overview of systematic reviews. *Evidence-based Complementary and Alternative Medicine: ECAM*.

[12] Shea B. J., Reeves B. C., Wells G. (2017). AMSTAR 2: a critical appraisal tool for systematic reviews that include randomised or non-randomised studies of healthcare interventions, or both. *BMJ*.

[13] Guyatt G. H., Oxman A. D., Vist G. E. (2008). GRADE: an emerging consensus on rating quality of evidence and strength of recommendations. *BMJ*.

[14] Li G., Zheng Q.-X., Liao Y.-T., Tan J.-Y., Xie Q.-L., Rask M. (2017). Effects of traditional Chinese exercises on the rehabilitation of limb function among stroke patients: a systematic review and meta-analysis. *Complementary Therapies in Clinical Practice*.

[15] Wang J. L., Dai X. J., Shi Q. (2020). Effectiveness of acupoint massage on post-stroke dysphagia: a meta-analysis. *Chinese Nursing Rrsearch*.

[16] Yue L. L., Li H. Y. (2019). Effectiveness of TCM emotional nursing on post-stroke depression: a systematic review. *Medical Frontier*.

[17] Li J. Y., Fang Z. Q., Xiao H. L. (2019). Meta-analysis of the effect of TCM nursing intervention for post-stroke constipation. *Journal of Guiyang University of Chinese Medicine*.

[18] Wu Q., Yang S. Y., Jia Y. (2018). Meta-analysis of effect of alternating hot-cold traditional Chinese medicine immersion for shoulder hand syndrome after stroke. *Chinese Evidence-Based Nursing*.

[19] Wu Q., Zhao L. M., Gong S. Q. (2018). Massage for swallowing disorders after stroke: a systematic review. *Journal of Clinical and Pathological Research*.

[20] Wang Y. R., Li Q., Xu D. Y. (2017). Meta-analysis of curative effect of auricular point sticking in traditional Chinese medicine nursing on constipation of patients with stroke. *Chinese General Practice Nursing*.

[21] Yuan A. L., Wang S. L. (2016). Meta-analysis on Chinese traditional medical nursing pathways in the curative effect of patients with stroke sequela. *Chinese Journal of Ethnomedicine and Ethnopharmacy*.

[22] Zhang J. Z., Liu K. (2016). Traditional Chinese medicine nursing on the rehabilitation of patients with cerebral apoplexy: a meta-analysis. *Journal of Liaoning University of Traditional Chinese Medicine*.

[23] Liang D., Zhang Y. F., Li M. (2015). Clinical efficacy on TCM emotional care to improve depressive symptoms in post-stroke depression patients: a systematic review. *Liaoning Journal of Traditional Chinese Medicine*.

[24] Peng L. L., Cheng F., Liu C. (2015). Systematic review s of characteristics TCM nursing in patients with stroke. *Chinese Medicine Modern Distance Education of China*.

[25] Taylor-Piliae R. E., Coull B. M. (2012). Community-based Yang-style Tai Chi is safe and feasible in chronic stroke: a pilot study. *Clinical Rehabilitation*.

[26] Au-yeung S. S. Y., Hui-Chan C. W. Y., Tang J. C. S. (2009). Short-form Tai Chi improves standing balance of people with chronic stroke. *Neurorehabilitation and Neural Repair*.

[27] Wang X. B., Hou M. J., Tao J. (2016). The effect of Tai Chi Yunshou on gait of community-based hemiplegic patients after stroke. *Chinese Journal of Rehabilitation Medicine*.

[28] Huang Y. D. (2016). The study of Taijiquan exercise on movement function and nerve excitability in patients with stroke.

[29] Wang X. Y. (2016). Effect of Tai Chi “Yunshou” training on balance function in patients with hemiplegia after stroke in community-based rehabilitation.

[30] Lv L. M. (2012). *Clinical Research of Magnetic Bead Aucupressure Auricular Point on Patients with Constipation after Stroke*.

[31] Lei Y. (2012). *Neck Acupoint Massage on Stroke Dysfunction after Intervention*.

[32] Wei Y. (2016). Efficacy of swallowing training combined with acupoint massage on stroke patients with dysphagia. *Journal of Nursing*.

[33] Zhao D. (2011). *Study on Supranuclear Paralysis Dysphagia with Stroke by Acupoint Pressure therapy*.

[34] Yao Q. L., Wu J. H., Zheng P. (2012). Rehabilitation effect of acupoint pressing on motor function and depression symptoms in patients with early stroke hemiplegia. *Journal of Beijing University of Traditional Chinese Medicine*.

[35] Hu Y. F. (2013). Effect of auricular point pressing on depression in patients with early stroke and hemiplegia. *Journal of New Chinese Medicine*.

[36] Yu C. X., Jin L. H., Chen X. F. (2014). Observation on the efficacy of auricular point pressing bean in the treatment of constipation in patients with traumatic subarachnoid hemorrhage. *Nursing and Rehabilitation Journal*.

[37] Ji J., Ren S. L., Wang Y. W. (2015). Observation on the efficacy of auricular point pressing in the treatment of empirical constipation in acute stage of cerebral apoplexy. *Beijing Journal of Traditional Chinese Medicine*.

[38] Cai B. C., Zhang C. Y. (2015). Therapeutic effect observation of otopoint-pellet pressure on improving constipation symptoms of stroke patients. *Chinese Journal of Modern Distance Education of China*.

[39] Guo Y. H., Chen H. X., Yang Z. J. (2011). Observation on the efficacy of alternating hot and cold traditional Chinese medicine immersion therapy combined with rehabilitation training in the treatment of shoulder-hand syndrome after cerebral apoplexy. *Chinese Journal of Physical Medicine and Rehabilitation*.

[40] Zhang P. J., Guo J., Bai Y. J. (2014). Observation on the therapeutic effect of modified alternating immersion therapy of hot and cold traditional Chinese medicine on shoulder-hand syndrome after stroke. *Journal of Clinical Research of Traditional Chinese Medicine*.

[41] Qu P. J. (2013). A randomized parallel controlled study on characteristic nursing of traditional Chinese medicine in convalescent patients with cerebral infarction. *Journal of Practical Traditional Chinese Internal Medicine*.

[42] Hao Y., Liu H., Yue S., Liu X. (2011). Introducing traditional Chinese nursing: a review of concepts, theories and practices. *International Nursing Review*.

[43] Zhao J.-Q., Zhou F., Sun Y., Tian R.-X., Adler-Collins J. K., Hao Y.-F. (2016). Insights on the development of TCM nursing. *International Journal of Nursing Sciences*.

[44] Shen Q. (2010). Factors and countermeasures affecting clinical application and development of traditional Chinese medicine nursing technology. *Chinese Journal of Nursing*.

[45] Rolfe G., Segrott J., Jordan S. (2008). Tensions and contradictions in nurses’ perspectives of evidence-based practice. *Journal of Nursing Management*.

